# Characterization of Atlantic Cod Spawning Habitat and Behavior in Icelandic Coastal Waters

**DOI:** 10.1371/journal.pone.0051321

**Published:** 2012-12-07

**Authors:** Timothy B. Grabowski, Kevin M. Boswell, Bruce J. McAdam, R. J. David Wells, Guđrún Marteinsdóttir

**Affiliations:** 1 Institute of Biology, University of Iceland, Reykjavík, Iceland; 2 U.S. Geological Survey, Texas Cooperative Fish and Wildlife Research Unit, Texas Tech University, Lubbock, Texas, United States of America; 3 Department of Oceanography and Coastal Sciences, Louisiana State University, Baton Rouge, Louisiana, United States of America; 4 Marine Sciences Program, Department of Biological Sciences, Florida International University, North Miami, Florida, United States of America; 5 Institute of Aquaculture, The University of Stirling, Stirling, United Kingdom; 6 Department of Marine Biology, Texas A&M University at Galveston, Galveston, Texas, United States of America; Technical University of Denmark, Denmark

## Abstract

The physical habitat used during spawning may potentially be an important factor affecting reproductive output of broadcast spawning marine fishes, particularly for species with complex, substrate-oriented mating systems and behaviors, such as Atlantic cod Gadus morhua. We characterized the habitat use and behavior of spawning Atlantic cod at two locations off the coast of southwestern Iceland during a 2-d research cruise (15–16 April 2009). We simultaneously operated two different active hydroacoustic gear types, a split beam echosounder and a dual frequency imaging sonar (DIDSON), as well as a remotely operated underwater vehicle (ROV). A total of five fish species were identified through ROV surveys: including cusk Brosme brosme, Atlantic cod, haddock Melanogrammus aeglefinus, lemon sole Microstomus kitt, and Atlantic redfish Sebastes spp. Of the three habitats identified in the acoustic surveys, the transitional habitat between boulder/lava field and sand habitats was characterized by greater fish density and acoustic target strength compared to that of sand or boulder/lava field habitats independently. Atlantic cod were observed behaving in a manner consistent with published descriptions of spawning. Individuals were observed ascending 1–5 m into the water column from the bottom at an average vertical swimming speed of 0.20–0.25 m s^−1^ and maintained an average spacing of 1.0–1.4 m between individuals. Our results suggest that cod do not choose spawning locations indiscriminately despite the fact that it is a broadcast spawning fish with planktonic eggs that are released well above the seafloor.

## Introduction

The physical habitat used during spawning is a frequently neglected aspect of the reproductive biology and output of marine broadcast spawning fishes. The underlying assumption is that since the eggs are quickly carried away from the site of release, the physical habitat at the spawning location does not make a substantial contribution to the survival of the progeny [1]. Research has instead focused on the qualities of the water column such as temperature, salinity, current patterns, etc. Unquestionably these characteristics of the water column are the major determinants of larval survival and dispersal, but the role of the physical habitat to overall reproductive output is uncertain. Fishermen have long targeted spawning aggregations of species that return annually to the same physical locations to spawn. For example, written accounts indicate that fishermen have been aware of the location and timing of Atlantic cod *Gadus morhua* spawning aggregations in northern Norway since at least the 9^th^ Century and these traditional fishing grounds are still exploited today [2]. Similarly in Iceland, the original Norse settlers quickly became familiar with the locations that yielded higher catches than others throughout the year [3]. The spawning sites investigated as part of the current study are among those described in manuscripts dating from the 14th Century [3]. This high level of spawning site fidelity is not unusual in Atlantic cod [4]–[8] and may be common amongst other broadcast spawning marine species [9]–[12]. Several reasons for this fidelity have been proposed ranging from cultural transmission [13] to an attraction to features that create suitable hydrographic conditions [14]–[16]. However, studies that characterize or quantify the habitat associations of broadcast-spawning marine fishes during reproduction are relatively uncommon and tend to be restricted to tropical reef fishes [16].

Atlantic cod is a broadcast spawning fish that releases buoyant eggs into the water column where they are fertilized and undergo development. Yet there is a large component of Atlantic cod reproductive behavior that seems to be tied to the substrate [17]–[18]. Cod mating systems have been characterized as a lekking system where males form aggregations on the bottom that are visited by females. Upon completion of a successful courtship, a male and female pair either release gametes near the substrate [17] or rise several meters into the water column to release gametes [19]–[23]. The spawning pair may be joined by a number of trailing males attempting to opportunistically fertilize some of the eggs [24]–[25]. Spawning Atlantic cod exhibit a great deal of variability in the habitat used for spawning in depth, current patterns, and general geographic locations, such as within fjord systems or on the continental shelf [5]. However, there has been little attempt to characterize the physical habitats used by the spawning aggregations. It is unknown how the physical habitat may impact reproductive output, if cod are as plastic in their selection of spawning habitat as they are in other aspects of their behavior, or if the availability of these habitats has the potential to limit cod populations. We employed a non-invasive, multiple technique approach to characterize the habitat of spawning cod and the associated fish assemblage off southwestern Iceland in April 2009. Secondarily, we were able to observe aspects of cod spawning behavior that have not been well documented in the field.

## Materials and Methods

### Ethics Statement

No specific permits or approvals were required for this work due to the lack of capture of or contact with the study animals. No privately owned or protected lands were accessed during the course of this study, nor were any protected species sampled.

### Study Area

**Figure 1:**
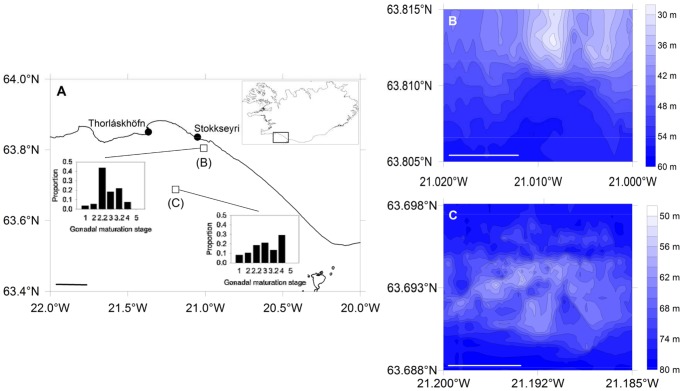
Map of study area off southwestern Iceland (A) with sample locations indicated by open boxes. Inset figures represent the proportion of cod exhibiting a particular gonadal maturity stage (1: immature; 2: maturing; 2.2: final stages of maturation; 3: ripe and running; 3.2: near spent; 4: spent/recovering; 5: omitted spawning) sampled from these sites during 17–20 April 2009 as part of the annual spring groundfish survey conducted by Hafrannsóknastofnunin. Scale bar represents 10 km. Bathymetric maps of Knarrarós (B) and Lofstadarhraun (C). Contour lines indicate 2-m isobaths. Scale bars in panels (B) and (C) represent 500 m.

We observed Atlantic cod aggregations on the submerged lava and boulder fields off the southwestern coast of Iceland (Figure 1). This area has been identified as the primary spawning grounds of the Icelandic cod stock [26]–[27]. Atlantic cod in reproductive condition are encountered on these primary spawning grounds off the southwest coast from March to early May and consistently aggregate year to year in well-defined locations [26]. Our study focused on the aggregations present at two locations: Knarrarós (63.810°N, 21.010°W), a submerged lava field located approximately 1.0 km offshore and 3.5 km SE of the town of Stokkseyri, and Lofstadarhraun (63.693°N, 21.192°W), another submerged lava field situated about 17 km offshore and approximately 20 km SSE of the town of Thorláskhöfn. References herein to inshore indicate data from Knarrorós, while offshore is in reference to Loftstadahraun. We focused on these sites because they have historically attracted spawning aggregations of Atlantic cod that tended to consist of larger individuals than other sites [26], [28]–[29] and data collected at these sites during annual gillnet surveys conducted by Hafrannsóknastofnunin (Marine Research Institute) during 17–20 April 2009 indicated the presence of actively spawning adults (A. Gunnarsson, Hafrannsóknastofnunin, pers. comm.; Figure 1). Macroscopic examination of the gonads indicated that approximately 80% of the cod captured at the inshore site (*n* = 55) and 50% of those from the offshore site (*n* = 38) were actively spawning or would be spawning imminently (stages 2.2–3.2). Water depth at the Knarrarós site ranged from 30–60 m and from 50–80 m at the Loftstadahraun site (Figure 1). Wind speed did not exceed 7 m s^−1^ during our survey, resulting in sea state conditions where wave heights were ≤0.5 m, uncharacteristically calm for mid-April in southwestern Iceland.

### Data Collection

Our behavioral observations were made during a 2-d research cruise (15–16 April 2009) aboard the R/V *Dröfn*. We operated two different active hydroacoustic gear types and a remotely operated underwater vehicle (ROV) simultaneously during the survey. A calibrated BioSonics DT-X split-beam echosounder (BioSonics, Inc., Seattle, Washington) equipped with a 120 KHz 6° split-beam digital transducer, operating at 0.4 ms, was used for bathymetric mapping, locating fish aggregations, and quantitatively describing the spatial distribution of the ensonified fish community. The transducer was mounted on a towfish deployed by crane off the port side of the vessel at approximately amidships. The towfish was flown approximately 2 m below the water surface. Using a crane at amidships on the starboard side of the vessel, we also deployed a DIDSON lens-based imaging sonar (Sound Metrics Corp., Lake Forest Park, Washington) mounted on a pan and tilt motor attached to a vane mount. The entire apparatus was suspended approximately 10 m above the substrate. The DIDSON pulses acoustic signals from a 96-beam transducer array at either 1.1 MHz, which utilizes 48 beams, or 1.8 MHz, which uses all 96 beams. Each beam has an effective angle of 0.3° vertical and 14° horizontal that produces a 28°×14° field of view when combined. The DIDSON was used to identify when cod were present, observe fish behavior and characterize habitat type. We used a VideoRay II ROV (VideoRay LLC., Phoenixville, Pennsylvania) to make observations of substrate composition and habitat type as well ground truth species identifications made using the DIDSON. The ROV was deployed as often as possible and coincident with the DIDSON and echosounder for multi-platform groundtruthing. The ROV was able to keep pace with the drifting vessel and provided for high-resolution substrate and fish community characterization. However, the ROV was not deployed at every sampling station due to difficulties maintaining control in fast currents among the channels of the lava fields and risking its tether becoming entangled or severed.

After arriving at a site, we deployed the split-beam echosounder to both map the habitat and locate fish aggregations. Upon location of an aggregation, identified by characteristic echogram patterns [19]–[22], we cruised approximately 300–500 m up current of that position and lowered the DIDSON. The ROV was deployed if conditions on the bottom were favorable for recovery. We drifted over the aggregation while simultaneously recording data from the DIDSON, split-beam echosounder, and ROV. Once we drifted over the fish aggregation and the lava field, the DIDSON and ROV were retrieved and we resumed our search pattern for fish aggregations.

### Data Processing and Analysis-habitat

**Figure 2:**
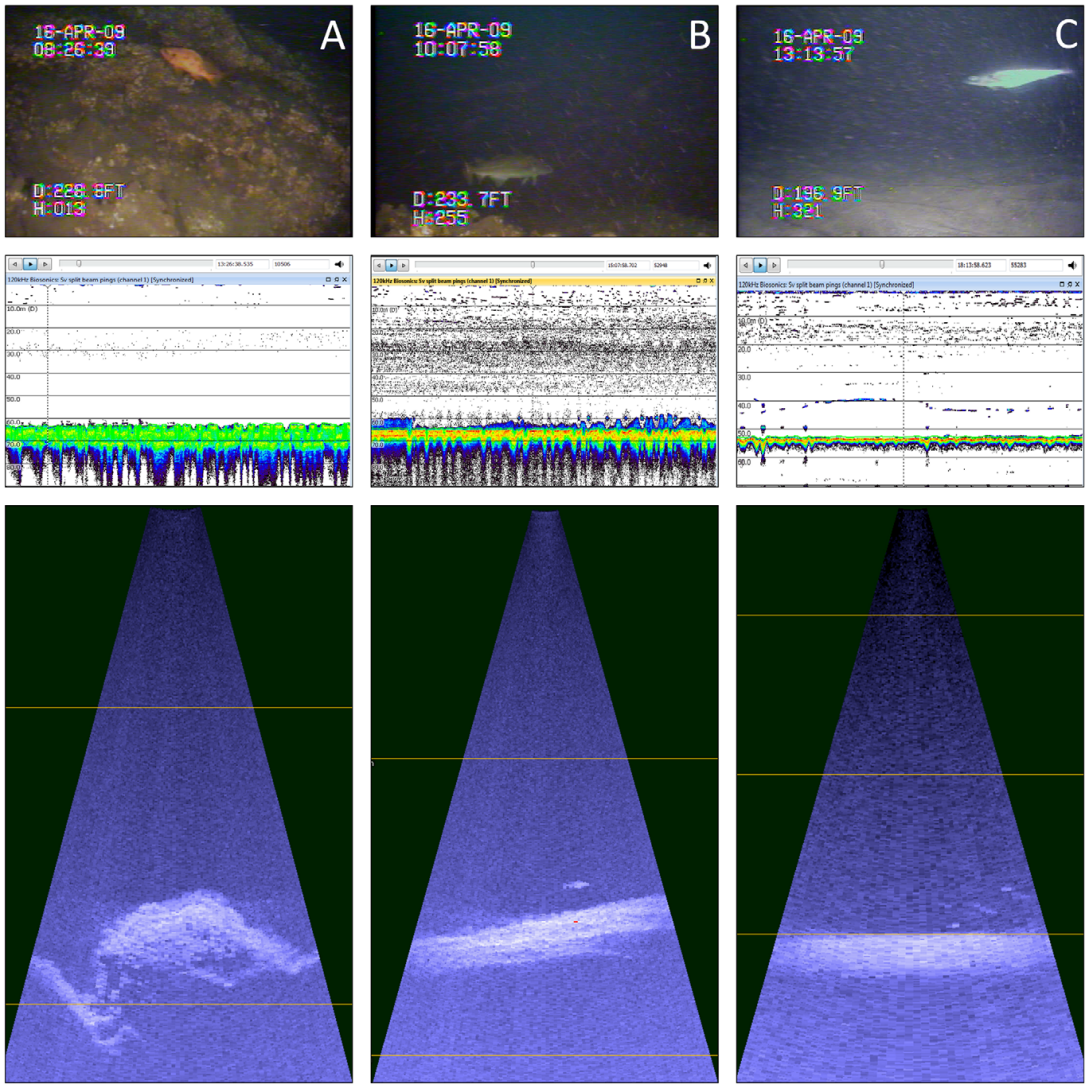
Example images collected from contemporaneously deployed ROV (top row), 120-kHz split beam echosounder (middle row), and DIDSON (bottom row) over boulder/lava field (a), transitional (b), and sand (c) designated habitats off southwest Iceland, 15–16 April 2009. Note redfish *Sebastes* sp. identified in ROV video data collected from boulder/lava field habitat, Atlantic cod *Gadus morhua* identified in both ROV and DIDSON data, and haddock *Melanogrammus aeglefinus* identified in the ROV data with faint targets detected near the substrate in the DIDSON and 120-kHz split beam echosounder data.

Habitat type was quantified by analyzing the video collected with the ROV. Analysis of the ROV video was performed in the laboratory by estimating the percent coverage of habitat type from 25 squares of equal size (9×9 cm) overlain on digital images of individual video frames. Percent coverage was divided among three categories including: (i) sand ridges; (ii) sand with intermixed rocky bottom; and (iii) boulder/lava fields (Figure 2). In addition, a maximum vertical-relief estimate, the maximum height (m) of any geological or biological structure within view, was made based on the change in depth of the ROV from seafloor to top of the structure.

Habitat-specific fish abundance was quantified for both ROV and DIDSON data using the *MIN/MAXIM* method [30], and fish were identified to the lowest possible taxonomic level. The minimum count (*MIN*), so-called because it is the minimum estimate of the number of species occurring in a transect, is the maximum number of a species observed within a single video frame. This method is commonly used for gregarious species, such as Atlantic cod, and is analogous to *MAXNO* [31], *MAX* [32], and *MaxN* [33]. Maximum counts (*MAXIM*) were also made to obtain total counts of each fish species seen over the entire video analyzed. Video counts of each species were modeled with a Poisson distribution. Specifically, a log-linear fixed effects model using the GENMOD procedure in SAS was used to predict fish numbers, with habitat as the independent factor [32], [34]. The model fit was evaluated with a maximum likelihood method and analysis of deviance. Dunn’s test was used to determine *a posteriori* differences among means (α = 0.05).

### Data Processing and Analysis-fish Distribution and Behavior

We used the display threshold and intensity settings contained in the SMC DIDSON control and display software package v. 5.25 (Sound Metrics Corp., Lake Forest Park, Washington) to optimize fish images, and the measuring tools to manually estimate the total length (TL) to the nearest cm of each fish within each frame. We also used the DIDSON the discriminate between fish species. Atlantic cod has a distinct size and shape compared to that of most other species potentially encountered at our study sites. However, two other species of gadoid fishes, pollock or saithe *Pollachius virens*, and haddock *Melanogrammus aeglefinus*, are common in the study area and similar enough to require specific criteria to discriminate them from Atlantic cod in the DIDSON images. Saithe were not expected to be present at high densities in the areas sampled because their spawning tends to occur earlier in the year (January–March) and in deeper water (100–200 m) than that of Atlantic cod [35]. Saithe also tend to be more streamlined than Atlantic cod and possess a forked caudal fin. Haddock overlaps in spawning season (April–May) and depth (50–200 m) with Atlantic cod [35] and does not show any clear morphological differences that would be apparent in the DIDSON images. However, adult haddock tend to be smaller than mature Atlantic cod. Based on the size distributions generated by gill net surveys in our sample sites and adjacent areas (Hafrannsóknastofnunin, unpublished data), individuals <50 cm were classified as haddock outright while individuals >60 cm were classified as Atlantic cod. Individuals 50–60 cm were classified as haddock unless they were part of an aggregation in which the majority of individuals was >60 cm.

**Figure 3:**
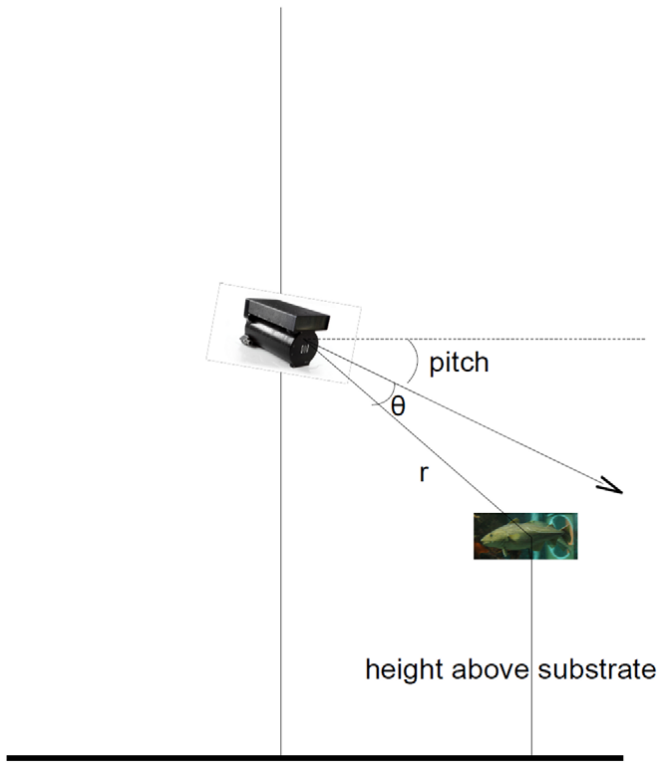
The dual-frequency identification sonar (DIDSON) views a horizontal plane of space, every point seen is described with a polar coordinate, (*r*, *θ)* within this (A). The direction of view (B) is expressed as three angles: yaw (compass angle with 0 degrees as north), pitch (or tilt, the angle up or down relative to horizontal, with 0 degrees horizontal and −90 degrees looking vertically down), and roll (displacement of the observed horizon from horizontal). First the polar coordinates (*r, θ)* are converted to (x, y) in meters relative to the DIDSON. (x, y) is then rotated in three dimensions using the rotation transformation matrices corresponding to roll, pitch and yaw in turn to get a location (depth relative to DIDSON and displacement north and east).

The SMC DIDSON control and display software package v. 5.25 (Sound Metrics Corp., Lake Forest Park, Washington) also was used to collect the data necessary to estimate the position of each fish relative to the bottom or each other. Fish position relative to the DIDSON was estimated by recording the measurements of range, *r,* and angle, *θ*, defined as the angle from the center-line of the field of view at which the fish was sighted, generated by placing the measuring tool on the anterior most visible portion of a fish. This process was repeated for each fish within each frame. We also recorded *r* and *θ* of the observed portion of the substrate in each frame for periods when the DIDSON was oriented vertically and close enough to the seabed. The range, *r*, and angle, *θ,* were combined with the direction of view of the DIDSON (i.e., the roll, pitch and yaw obtained from its onboard compass and tilt sensors), to transform these polar coordinates into a Cartesian form of meters depth relative to the DIDSON and meters north and east. This is an application of rotational transformation matrices as in Figure 3. Where substrate was visible, the height of the DIDSON above it was calculated using the same method and the fishes’ vertical positions were converted to height above seabed. For each fish visible in every frame, we calculated the total distance and vertical component of distance to every other fish in that frame, and recorded the identity and distance to its nearest neighbor. When a fish was visible for several frames, its vertical speed (rate of ascent or descent) was calculated.

Processing of the split-beam echosounder data was accomplished using Echoview 5.2 (Myriax Pty. Ltd., Hobart, Australia). Analysis thresholds were applied to all along-transect S_V_ (volume backscatter; −80 dB re 1 m^−1^) and target strength (−55 dB) echograms. Calibration settings were applied to compensate for temperature and salinity effects on sound speed attenuation based on temperature and salinity profiles. Following parameter configuration, echograms were visually inspected for bad data regions (i.e., abnormal towfish behavior or loss of signal) and manually excluded. Data within 3 m of the transducer face were excluded to account for surface noise and nearfield effects. A bottom detection algorithm with a 0.5 m backstep [36] was applied to exclude the sea floor and boulder/lava field structure from the analysis, and then manually edited. Along-transect areal backscatter densities (s_A_t, m^2^ nmi^−2^) [37] were classified by habitat type (sand ridges, sand with intermixed rocky bottom [transition zone], and boulder/lava fields) and exported from Echoview in 50-m horizontal×10-m vertical cells. s_A_ is a widely-used measure of relative backscatter in the absence of robust echo-trace classification and target strength estimates [38]–[39], with the assumption that gross changes in biomass distribution will be detectable against the backdrop of varying target types and their associated target strengths. Habitat type and transitions were characterized by ROV and DIDSON collections as described below.

*In situ* target strength estimates of detected single targets (TS_ST_) were classified by habitat type and used to scale the s_A_ values to target density (targets nmi^−2^). Single targets were identified using a split-beam single target detection algorithm (method II) within Echoview where targets fulfilling single target criteria with target strength greater than −55 dB [40] were accepted into the analysis, corresponding to a target approximately 3.2 cm in length [41], following the implicit assumption that fish length is proportional to TS_ST_ [39]. The single target algorithm was tuned to accept targets with echo envelopes between 0.6 and 1.7 times the pulse length, with a maximum beam compensation of 12 dB. In areas where targets were too dense to discriminate individuals, the single targets along the periphery of the aggregation were assumed to represent those within the aggregation. This assumption was supported with complementary data from the DIDSON. Target strength values of isolated targets assumed to be individual fish were used to scale integrated backscatter measures to derive an estimate of fish density (*p*; fish nmi^−2^) for each elementary sampling distance unit (EDSU) using the following equation:
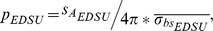
where 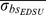 represents the mean backscattering cross section (m^2^; the linear equivalent of target strength) of single targets within each analysis cell [37].

Habitat-types were classified into three categories, sand ridges, sand with intermixed rocky bottom [transition zone], and boulder/lava fields, according to the ROV and DIDSON imagery. When ROV and DIDSON were not available, habitats were identified by acoustic properties of the substrate types. Transition zones were defined as the interface between sand and boulder/lava field habitats and included a 50 m buffer on either side of the substrate type.

Variability in along-transect EDSU-specific s_A_ and TS_ST_ across habitats were analyzed separately using a general linearized mixed model (GLIMMIX) [42] to test for the effects of habitat type and depth with each EDSU representing an observation. The GLIMMIX models were preferred for fitting data that are non-normally distributed [43]; where the distributions are specified (e.g., lognormal) and fit to the raw data. We fitted *p* and TS_ST_ to a negative binomial distribution. Prior to statistical analyses, TS_ST_ values were linearly transformed [37]. In all ANOVA models, the residuals were tested for normality and Tukey’s honestly significant difference (HSD) post hoc test was used to identify differences in means among pairwise comparisons. All means are reported as least-squares means, and estimates of error are represented by upper and lower 90^th^ percentile confidence interval derived from the LSmeans estimates.

## Results

### Habitat Characterization and Use

Habitat type surveyed by each gear type varied over the study period. Total habitat-specific effort using ROV over sand ridges was 115 minutes (38.1% total survey time), 9 minutes over transition zones (3.0% total survey time), and 178 minutes over boulder/lava fields (58.9% total survey time). Maximum vertical-relief estimates for each habitat type quantified by ROV surveys averaged 10 cm over sand ridges, 1 m over sand with intermixed rocky bottom, and 2 m over boulder/lava fields. In addition, DIDSON effort was more evenly distributed over two habitats: sand habitat effort totaled 193 minutes (42% total survey time) and boulder/lava fields consisted of 267 minutes (58% total survey time).

Five fish species, cusk *Brosme brosme*, Atlantic cod, haddock, lemon sole *Microstomus kitt*, and Atlantic redfish *Sebastes* spp., were identified through ROV surveys. While the relative habitat-specific abundance of four of the five species identified through ROV surveys suggested the highest numbers were present over the boulder/lava field habitat, only *Sebastes* spp. showed a significant habitat effect (*P*<0.001) with an average abundance of 0.32 min^−1^. Relative abundance estimates of the three other species, though non-significant, over boulder/lava field habitat averaged 0.02 min^−1^ for cusk, 0.05 min^−1^ for cod, and 0.10 min^−1^ for haddock. Lemon sole had greater numbers over sand ridge habitat, averaging 0.02 min^−1^, but again the habitat-specific differences were not statistically significant (*P = *0.965). No fishes were observed over the transitional habitat. Results from DIDSON surveys indicated relative abundance of fish were four times higher over boulder/lava field habitat (3.97 min^−1^) compared to sand habitat (0.93 min^−1^; P<0.05).

**Figure 4:**
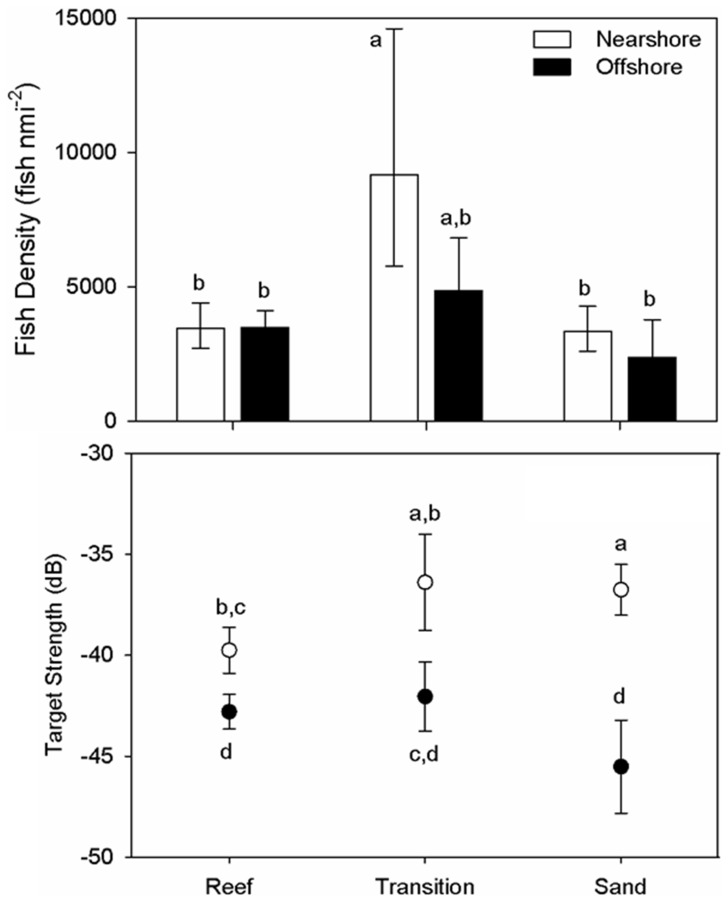
Least-square means estimates of acoustic fish density (fish nmi^−2^; upper panel) and mean acoustic target strength; lower panel) of fish at boulder/lava field (reef), sand, and transitional habitat surveyed off southwestern Iceland 15–16 April 2009 using a split beam echosounder with a 120 KHz 6° split-beam digital transducer operating at 0.4 ms. Nearshore stations (white) and offshore (filled) stations are illustrated. Error bars represent upper and lower 90^th^ percentile confidence intervals.

Of the three habitats identified during the surveys, the transition areas between boulder/lava field and sand habitats were characterized by significantly greater fish density (6672.6 fish nmi^−2^, LCL = 5005.6, UCL = 8894.6; *P*<0.001) than compared to boulder/lava field (3480.4 fish nmi^−2^, LCL = 3012.1, UCL = 4021.6) and sand habitats (2819.2 fish nmi^−2^, LCL = 2169.4, UCL = 3663.8; Figure 4). We observed greater than a 30% increase in fish density at nearshore stations (4734.7 fish nmi^−2^, LCL = 3903.1, UCL = 5743.7) than offshore (3430.1 fish nmi^−2^, LCL = 2814.6, UCL = 4182.1), with nearshore transition zone contributing to the greatest contrast among habitat levels (Figure 4).

Estimates of pairwise comparisons of target strength followed trends similar to those observed with fish density. The transition zone between habitats comprised of significantly larger targets (−38.0 dB, UCL = −36.8 dB, LCL = −39.3 dB) than the boulder/lava field (−41.3 dB, LCL = −42.1 dB, UCL = −40.6 dB; *P*<0.02) and approximately the same size as those over sand (−41.1 dB, LCL = −42.5 dB, UCL = −39.8 dB; *P = *0.14) habitats. Nearshore habitats had consistently larger targets (by 6 dB; *P*<0.001) than offshore habitats. As with the fish density, target strength was significantly greatest at the nearshore transition zone by at least 3 dB (*P*<0.001; Figure 4), except for the nearshore sand (*P* = 0.79).

### Atlantic Cod Behavior

**Figure 5:**
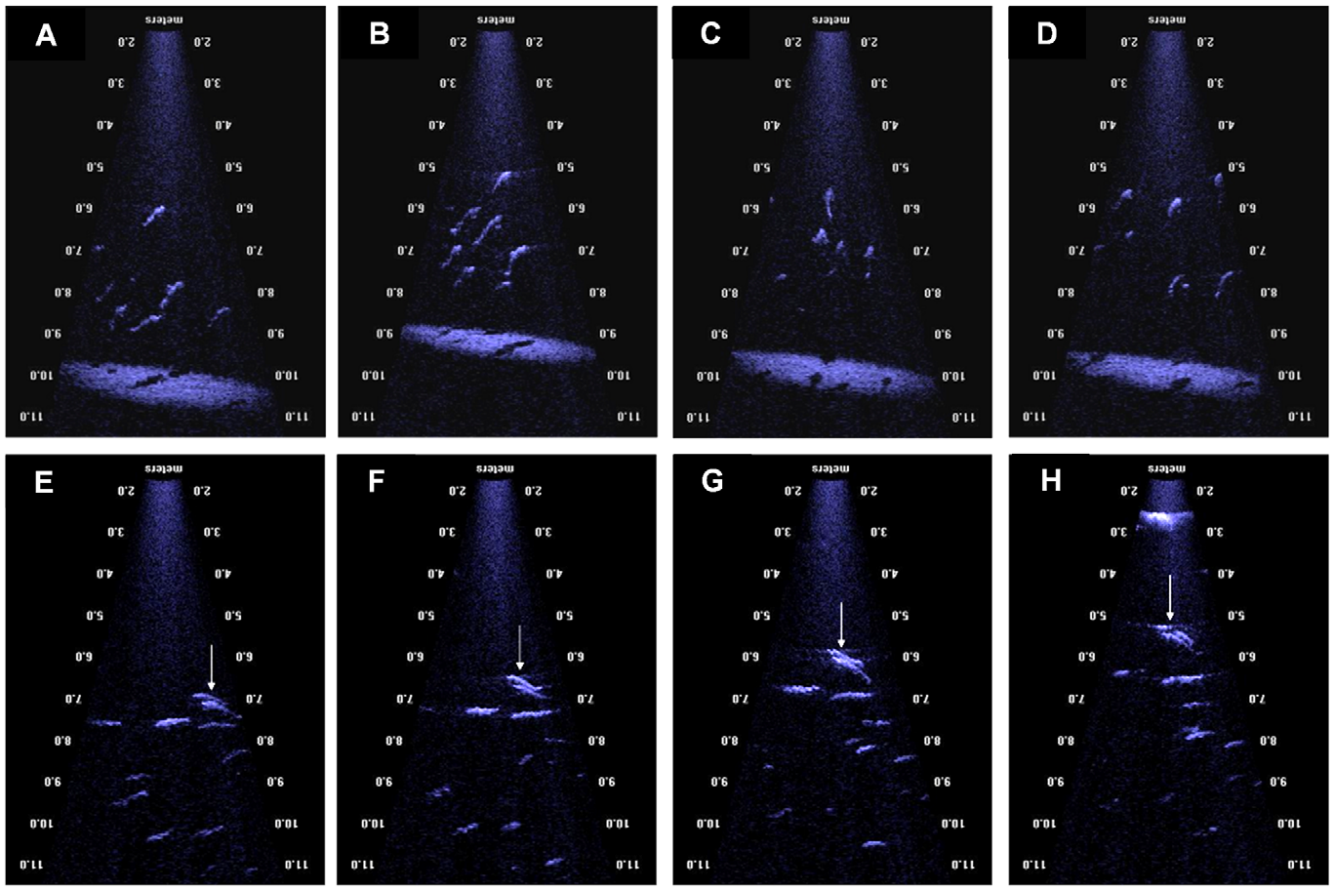
Images of Atlantic cod captured by a dual-frequency identification sonar (DIDSON) deployed approximately 1.0 km off the coast of Iceland at Knarrarós on 15 April 2009. In panels a–d, the DIDSON is positioned approximately 10 m off the bottom and is oriented at a 30° angle off perpendicular to the substrate. The Atlantic cod shown in these panels (a–d) are swimming up into the water column. The DIDSON is the same distance above the substrate in panels e–h, but oriented at approximately a 60° angle to it. These cod seem to have reached the depth that the fish in panels a–d were ascending to and are swimming parallel to the substrate. Arrows indicate two individuals that may be coupling and engaged in a spawning event. The full video file from which these images were captured can be found in the supplementary information (Video S1).

**Figure 6:**
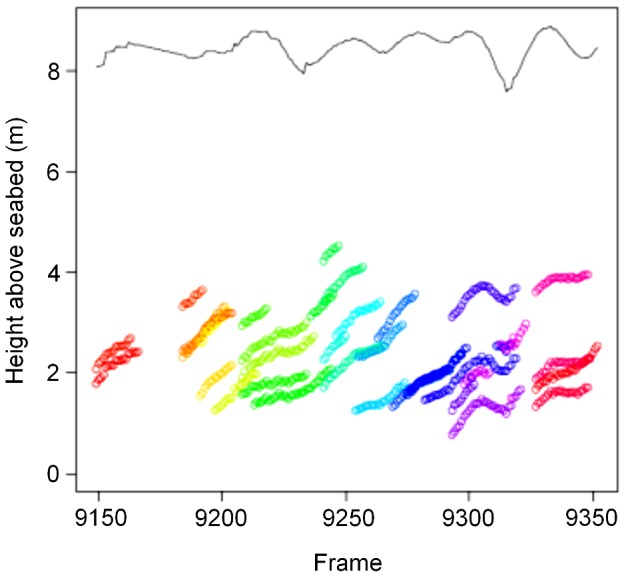
Plot of height above the seabed of individual Atlantic cod in a presumptive spawning column over time as determined using a dual-frequency identification sonar (DIDSON) deployed off southwestern Iceland 16 April 2009. Different colors represent individual fish. Figure describes the positions of fish seen in Video S1.

Images of cod aggregations were captured by the DIDSON at two distinct times on 15 April 2009 (see Video S1). The first set of images encompasses 33 seconds, comprising 238 data frames, starting at 13∶49 GMT when the DIDSON was oriented at approximately a 30° angle from perpendicular to the substrate. During this segment, we observed 34 cod ranging from 63−110 cm in total length (mean ± SD: 83±11 cm) and as many as six individuals in a single data frame. These cod were swimming 2.4±0.7 m (mean ± SD) above the sea bed (Figure 5). We were unable to calculate an absolute swimming speed for individual cod due to movements of the DIDSON. They were ascending into the water column at mean vertical swimming speed of 0.25±0.12 m s^−1^ (Figure 6a–d). Individuals maintained a mean (± SD) distance of 1.0±0.4 m between themselves and their nearest neighbor, and this spacing was not correlated to the total length of the individuals involved (linear regression of distance against total length; *P* = 0.17). However, while body size did not seem to influence spacing, larger individuals did seem to be swimming to greater heights in the water column than their smaller counterparts (linear regression of distance against total length and time; *R* ^2^ = 0.13; *P*<0.001).

At 13∶50 the DIDSON was tilted to approximately a 60° angle from perpendicular to the substrate to follow the cod aggregation. An additional 599 data frames were captured encompassing 86 seconds. During this second observation period, cod were observed swimming approximately 4.5±1.2 m (mean ± SD) above the substrate (Figure 6e–h). The 51 cod that we observed during this second observation period ranged from 57–127 cm in total length (mean ± SD: 89±16 cm). We observed as many as 10 cod in a given data frame simultaneously. The observed fish were ascending at approximately the same rate as individuals observed in the first period, though five individuals were descending (mean ± SD = 0.20±0.19 m s^−1^; range = −0.63–0.33 m s^−1^). There was no relationship between the size of an individual and the distance to its nearest neighbor (linear regression; *P = *0.30), as seen in the individuals during the first observation period. Cod during this second observation period also maintained similar spacing to that seen amongst individuals during the first observation period (mean ± SD = 1.3±1.1 m).

We also observed what appeared to be two individuals coming together and staying in very close proximity for several seconds on two occasions during this second observation period. One of these events is indicated by arrows in Figure 6e–h. These two fish were 118 cm and 97 cm TL and positioned 0.24±0.07 m (mean ± SD) apart for approximately 2 s (15 frames). The other event consisted of two individuals (105 cm, 88 cm) swimming approximately 0.33±0.12 m (mean ± SD) apart for 6 s (61 frames). It is not entirely clear what is occurring in these frames as it is difficult to ascertain whether the two individuals are in the same plane from this perspective, but their approach and orientation seems\ consistent with previous accounts of cod coupling and spawning behavior [17]–[18], [25], [44].

## Discussion

**Figure 7:**
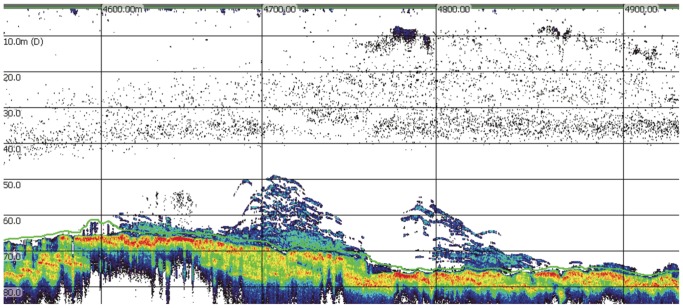
Echogram of cod spawning aggregation in the transitional habitat between boulder/lava field (left) and sand (right) on Lofstadarhraun off southwestern Iceland 16 April 2009.

The tendencies of structurally complex habitats and transitional habitats between ecotones to attract disproportionately higher fish biomass and biodiversity than surrounding habitats, are both well-documented phenomena [45]–[46]. Our data suggest that this is also the case for the boulder/lava fields and surrounding transitional habitats off southwestern Iceland. The structurally complex boulder/lava fields and the transitional habitat between them and sandy bottomed habitat supported higher fish densities relative to that of the surrounding sandy-bottom habitat despite comprising a relatively small proportion of the available habitat surveyed. The boulder/lava fields appear to be the preferred habitat of *Sebastes* spp., which is consistent with previous descriptions of these species having an association with structurally-complex habitats [35], [47]. Our data also suggested that the boulder/lava fields and the edge habitat surrounding them might be the preferred habitat for cusk, haddock, and cod, but the limited number of direct observations of these species by the ROV did not conclusively demonstrate this habitat use pattern. The split-beam echosounder data indicated both a higher total density of fish along the edge habitats surrounding the boulder/lava fields and larger individual fish (Figure 7). The DIDSON data collected from these areas suggested these fish observed by the split beam echosounder were gadoids based on body shape and size and confirmed that these individuals were present in relatively dense shoals. These dense shoals of fish were not observed over the surveyed sand bottom habitat. Furthermore, gillnet surveys in our study areas by the Icelandic Marine Research Institute conducted within 4–7 days of our survey indicated the presence of both Atlantic cod and haddock with distinct length-frequency distributions (A. Gunnarsson, Hafrannsóknastofnunin, pers. comm.). However, the potential limitations of using size to discriminate between species must be noted. It is possible that smaller cod, particularly juveniles which may linger in areas adjacent to spawning aggregations [21], might be misclassified using the size criteria established in this study in cases where supporting video data was unavailable. While this could potentially result in an underestimate of the biomass and scope of habitat use of Atlantic cod, it would not impact our primary conclusions focused on the habitat occupied by adult cod.

While there is the potential that this high abundance of gadoid fishes is a result of an edge effect, several lines of evidence suggest that cod presence may have been a function of reproductive activity. Spawning gadoids have been targeted both in this general region and at these specific sites for the last 500 years or so [3]. Also fishery logbook data and routine sampling from landed catch demonstrate high frequencies (>80–90%) of spawning fish in the area during the spawning season [26], [48]. The gillnet surveys conducted only a few days prior to our survey at and around our study sites showed that nearly all the cod captured were either spawning or spent (stage-III–IV; A. Gunnarsson, Hafrannsóknastofnunin, pers. comm.). We were unable to make direct observations of large numbers of cod using the ROV. However, the large shoals of fish interpreted as cod were consistent with the descriptions of cod spawning aggregations from the literature [19]–[22] and the DIDSON data revealed behavioral patterns consistent with that reported for spawning cod.

The Atlantic cod observed in the DIDSON images may have been participating in what has been described as a “spawning column” [19]–[22]. Rose [19] surveyed cod spawning aggregations off Newfoundland and Labrador with sonar and described groups of cod ascending up to 50 m above the main body of an aggregation situated on the seafloor at approximately 350–375 m depth. These groups appeared in the sonograms as distinct columns. Observations of spawning cod in shallower waters (30–50 m) indicate that these spawning columns may take on a more layered appearance in echograms [20]–[22], though it is unclear whether this is due to differences in methodology or in cod behavior. The formation of spawning columns has not been noted in all cod populations [49], nor in captive studies of cod spawning behavior [17], [44], suggesting differences amongst populations [20]–[22] or behavioral plasticity, though relatively shallow tanks may restrict vertical movement in captive studies. The aggregations observed in this study were more similar in appearance to the layered columns noted by Fudge and Rose [20] (Figure 7).

The rate of ascent of individual cod within their spawning columns has not been previously described. Our estimates of 0.20–0.25 m s^−1^ (12–15 m min^−1^) are considerably higher than the maximum ascent rates of 1–3 m min^−1^ previously reported for this species [50]–[51]. However, these previously reported ascent rates were estimated from tagged cod outside of their spawning season while making vertical movements of 10 s to 100 s of meters over the course of minutes to hours [50]–[51]. The cod in the spawning columns seemed to be quickly ascending no more than 5–10 m into the water column. It is not clear how long the individuals remained at these depths before returning to the seafloor, but evidence from various sources suggest that the entire reproductive sequence from courtship to gamete release lasts only a few minutes [17], [52]–[54]. Given the short duration of time an individual seems to spend in a spawning column, it is unlikely that these cod make any significant adjustments to their swim bladder [50], [55]. Furthermore, it is possible that individuals maintain negative buoyancy while in the spawning aggregation to compensate for the rapid ascent rate while participating in a spawning column and reduce the risk of an uncontrolled ascent due to an overexpansion of gas in the swim bladder [50], [55].

In general, the cod within presumptive spawning columns seemed to maintain a spacing of about a meter from their nearest neighbor. While we were unable to find other reports on the spacing of individuals within spawning columns, this spacing is similar to that maintained by individuals within the main body of a spawning aggregation [19]. However, the lack of relationship between the size of an individual and the distance to its nearest neighbor was unexpected. Size relationships are an important factor in the mating system and reproductive success [24], [44], [52], [56]–[57]. Generally, larger males experience greater reproductive success than their smaller counterparts [24], [52], [57], but mating tends to be size assortative, that is females are thought to prefer males of similar or slightly larger size [24], [44], [56]. However, a significant component of the reproductive output of a given spawning event can be attributed to the contribution of trailing males that follow a spawning pair while releasing gametes [24]–[25]. Spawning columns have been hypothesized to contain primarily male-female pairs of spawning cod [19] that leave the main aggregation on the seabed to avoid these opportunistic, trailing males [20]. However, the lack of any size-structuring in the observed spawning columns and the relatively low number of potential spawning events observed in this study suggest that cod spawning columns may be comprised primarily of opportunistic males trailing one or more actively spawning pairs.

We observed only two pairs of cod that were in close proximity to one another and exhibiting behavior and orientation that was consistent with published descriptions of cod courtship and spawning in captivity [17], [24]–[25], [44]. There were other aspects of the interaction between these pairs that support our conclusion that these individuals were engaged in spawning. As previously mentioned, the entire progression from courtship to gamete release requires only a few minutes and the process of gamete release is only a very small component of this progression (mean duration ± SE: 9.9±2.8 s; [17]). While the duration of the observed events were consistent with that reported in the literature, the events occurred at a greater distance from the substrate than that reported in other studies. Most studies report cod reproduction occurring within a few meters of the bottom based on the depth of capture of males in reproductive condition [17]–[18], [24]. However, some studies suggest that spawning may occur farther off the bottom [19]–[23]. Our data support the conclusion that while the main body of the spawning aggregation may be in close proximity to the seafloor, at least some of the gamete release may occur in the water column. Laboratory studies suggest that females show a preference for spawning with males that are the same size as or slightly larger than themselves [24]–[25]. The observed size difference between the two individuals in each pair was within the 5–25 cm range reported by Bekkevold et al. [24]. Unfortunately, the DIDSON did not provide images of sufficient resolution to determine if gamete release had occurred during these events to confirm our interpretation of these two pairs of cod. Furthermore, the orientation of the transducers relative to the cod did not allow for a definitive determination as to whether the two individuals are in the same plane. However, if our interpretation of these events is correct, it suggests that gamete release may occur throughout a spawning column and not solely at its apex. This may have implications on the modeling of egg and larval drift patterns depending on the prevailing oceanographic conditions of the spawning grounds.

Despite the fact that Atlantic cod is a broadcast spawning fish with planktonic eggs that are released well above the seafloor, our results suggest that cod do not chose spawning locations indiscriminately. Indeed, numerous studies have noted high-levels of fidelity of cod to specific spawning grounds [4], [6]–[8]. Our study was not sufficient in scope to address whether the degree of fidelity individual cod may exhibit to specific patches of edge habitat surrounding boulder/lava fields off southwest Iceland, nor was it able to evaluate any potential benefits conferred on the progeny of the adults spawning there. While these evolutionary and ecological implications of spawning site fidelity are interesting in their own right, the significance of a broadcast spawning fish having distinct habitat preferences during spawning should not be overlooked as the reasons for cod aggregations to form in the transitional habitats surrounding boulder/lava fields may have important conservation and management implications. For example, the boulder/lava fields on the Icelandic continental shelf are high relief structures that may serve as a landmark or focal point for cod spawning aggregations to coalesce around. Alternatively, spawning cod may benefit from being near these structurally complex habitats by taking advantage of how they may alter prevailing currents to aid in the dispersal or retention of early-life history stages or using them as refuges from strong tidal currents. Further research is necessary to understand the importance of benthic habitats to broadcast spawning marine fishes and how their populations might respond to changes in the quantity or quality of these habitats. Our results suggest that protecting the integrity of the habitat used by these spawning aggregations warrants consideration in conservation and management planning along with the more conventional focus on guarding against overexploitation of the aggregations of adults during the spawning season.

## Supporting Information

Video S1 **Video of Atlantic cod captured by a dual-frequency identification sonar (DIDSON) deployed approximately 1.0 km off the coast of Iceland at Knarrarós on 15 April 2009.** The DIDSON is positioned approximately 10 m off the bottom fur the duration of the video. During the initial part of the video, the DIDSON is oriented at a 30° angle off perpendicular to the substrate and later shifts to a 60° angle off perpendicular.
(WMV)
